# Glycemic control and COVID-19 outcomes across the continuum of glucose regulation: a retrospective cohort study

**DOI:** 10.3389/fcdhc.2026.1862850

**Published:** 2026-10-14

**Authors:** Armand Mbanya, Serena Leung, Alan Bienvenida, Elizabeth Leslie, Sangmin Ryan Shin, Kate E Koplan, Felipe Lobelo

**Affiliations:** 1 Department of Quality and Patient Safety, The Southeast Permanente Medical Group, Kaiser Permanente, Atlanta, GA, United States; 2 Hubert Department of Global Health, Rollins School of Public Health, Emory University, Atlanta, GA, United States

**Keywords:** COVID-19 outcomes, diabetes, glycemic control, HbA1c, hospitalization, mortality, prediabetes

## Abstract

**Introduction:**

Coronavirus disease 2019 (COVID-19) has disproportionately affected individuals with underlying metabolic dysfunction. This study investigates the association between varying levels of pre-infection glycemic control and COVID-19-related health outcomes.

**Methods:**

A retrospective chart review was conducted on Kaiser Permanente Georgia patients with confirmed COVID-19. Participants (N = 2071) were categorized into five groups based on their HbA1c levels: patients without diabetes, those with prediabetes, and those with diabetes with varying degrees of glycemic control. We adjusted for demographics, social determinants of health, comorbidities, medications, and lifestyle factors.

**Results:**

Among 2071 patients, 986 (47.6%) were without diabetes, 658 (31.8%) had prediabetes, 183 (8.8%) had diabetes with HbA1c<7%, 162 (7.8%) had diabetes with HbA1c ≥ 7% ≤ 9% and 82 (4%) had diabetes with HbA1c>9%. Patients with HbA1c>9% had the highest proportion hospitalized (38.3%, p<0.001), and admitted to ICU (14.8%, p<0.001). In multivariable logistic regression models, prediabetes and insulin use were associated with greater odds of hospitalization; odds ratio 1.43 [95% CI 1.09-1.87] and 3.02 [95% CI 1.95-4.68], respectively.

**Conclusions:**

There is a gradient of increasing COVID-19 severity with worsening glycemic control. The study shows the importance of recognizing prediabetes as a significant risk factor for severe COVID-19, comparable to poorly controlled diabetes.

## Introduction

Although COVID-19 has been declared to no longer be a public health emergency of international concern, it continues to impact global populations’ health profoundly. The United States has faced significant challenges, particularly among populations with underlying medical conditions (1, 2). The interplay between COVID-19 and chronic health issues has been a focal point of recent research, drawing attention to the heightened vulnerability and increased morbidity of these large segments of the population (1).

Central to these discussions is the link between altered glucose metabolism, including prediabetes and diabetes, and the severity of COVID-19 illness (3–5). Studies have consistently shown that individuals with diabetes, particularly type 2, are at an increased risk of severe COVID-19 complications and mortality (3). For instance, a recent comprehensive review by D’Souza et al. highlighted that patients with diabetes are more likely to experience severe symptoms, require hospitalization, and face higher mortality rates compared to individuals without diabetes (3). This connection underscores the importance of effective glucose control in ameliorating COVID-19 risk. Poor glycemic control, observed in both chronic diabetes and acute stress-induced hyperglycemia, has been linked to worse outcomes in COVID-19 patients (6). Furthermore, research by Sardu et al. indicates that patients with pre-existing diabetes and acute hyperglycemia at hospital admission for COVID-19 had worse outcomes compared to those with stress hyperglycemia without diabetes (7). Research by Liontos et al. shows that hyperglycemia upon hospital admission is a significant predictor of mortality in COVID-19 patients, irrespective of a diabetes diagnosis, while Marfella et al.’s work further indicates that optimal COVID-19 immunotherapy is not observed while patients have hyperglycemia, emphasizing the need for vigilant glycemic monitoring and management in this patient population (8, 9).

The increased disease risk for patients with cardiometabolic conditions such as hypertension and diabetes is demonstrated at the cellular level, as well. Research indicates that the entry and replication of SARS-CoV-2 are facilitated by transmembrane serine protease (TMPRSS2) and the angiotensin-converting enzyme 2 (ACE2) receptor, and dysregulation of these pathways such as in patients with cardiometabolic conditions have been linked to worse clinical outcomes (10, 11). Additionally, clinical data have indicated that patients with pre-existing cardiometabolic conditions, including hypertension and diabetes, are at higher risk of severe COVID-19 outcomes; these pre-existing conditions have been associated with higher hospitalization rates, ICU admission, and mortality, indicating the influence of comorbidities in patient prognosis (7, 12). Research also suggests that SARS-CoV-2 may cause endothelial dysfunction and hypercoagulation hence increasing risk of adverse cardiac events in COVID-19 patients (13).

Despite the established relationship between diabetes and COVID-19, there remains limited investigation on how the continuum of glucose regulation at a population level prior to infection, influences COVID-19 outcomes (8, 14). Given the substantial portion of the U.S. population affected by glucose dysregulation, especially prediabetes (1 in 3 adults), understanding its role in COVID-19 is crucial (15). Our study seeks to bridge this knowledge gap by comparing COVID-19 outcomes across the clinical spectrum of glycemic control, from normoglycemia through prediabetes to established diabetes. While previous studies have established the link between poor glycemic control and COVID-19 severity, our study uniquely examines this relationship across the entire spectrum of glycemic control and investigates the impact of pre-infection glycemic status on COVID-19 outcomes. This study utilizes retrospective chart review and pre-existing laboratory values which may limit causal inference due to potential incomplete data and residual bias, however it provides valuable real-world insights into the relationship between glycemic control and COVID-19 outcomes across a large and diverse population. This research aims to enhance our understanding of COVID-19 in the context of varying glucose control and inform targeted interventions for at-risk populations.

## Subjects

We conducted a retrospective analysis of patients affiliated with the Kaiser Permanente Georgia (KPGA) diagnosed with COVID-19 between March 3, 2020, and September 11, 2020. This cohort was formed by identifying all KPGA members with a laboratory-confirmed COVID-19 diagnosis during the specified period. KPGA is an integrated healthcare system serving the metro Atlanta area, providing a comprehensive view of the patient population in this region. This analysis primarily focused on categorising patients into five groups based on their HbA1c levels within 12 months of diagnosis. These groups included normo-glycemic patients without diabetes (HbA1c ≤ 5.6% (38 mmol/mol)), patients with prediabetes (HbA1c between 5.7% (39 mmol/mol) and 6.4% (46 mmol/mol)) patients with well-controlled diabetes (HbA1c < 7% (53 mmol/mol)), aligned to the recommended glycemic goal for many nonpregnant adults without severe hypoglycemia or hypoglycemia affecting health or quality of life, patients with moderately-controlled diabetes (HbA1c ≥ 7% (53 mmol/mol) ≤ 9% (75 mmol/mol)), and patients with poorly-controlled diabetes (HbA1c > 9% (75 mmol/mol)), consistent with the Healthcare Effectiveness Data and Information Set (HEDIS) definition of poor diabetes control (16, 17). The use of HbA1c levels within 12 months before COVID-19 diagnosis was chosen to reflect the patients’ glycemic status leading up to their infection, providing insight into how pre-infection glycemic control influences COVID-19 outcomes.

## Materials and methods

### Socio-demographic data collection

The socio-demographic profile of each COVID-19-confirmed patient was carefully documented. This included age, gender, self-reported race, type of insurance, and household median income. Education level and geographic area of residence within the metro Atlanta region were also recorded. For a more comprehensive understanding of the social determinants of health (SDOH), we utilized the neighborhood deprivation index (NDI), along with ESRI^®^ Business Analyst data (18, 19). This approach allowed us to interpolate socioeconomic status using medical record numbers (MRN) linked to zip code classifications for median household income and educational attainment.

### Identification of risk factors

We extracted the comorbidity profile of each patient from their medical history and clinical diagnoses using the International Statistical Classification of Diseases and Related Health Problems codes (ICD-10) (20). The Charlson Comorbidity Index (CCI) was employed to measure the overall comorbidity burden (21). Pharmacy dispensing data was used to ascertain the frequency of outpatient medications. The HEDIS was a tool for evaluating nephropathy management and eye care (22). Blood pressure control was defined based on the most recent reading within the last 12 months, <140/90 mmHg. Additionally, patients’ exercise habits were documented, categorizing those with less than 10 minutes of weekly exercise as inactive.

### Clinical outcomes assessment

The primary focus was on various clinical outcomes for hospitalized patients with confirmed COVID-19. This included the average length of hospital stay, admissions to intensive care unit (ICU) and the duration of stay, use of mechanical ventilation, and mortality rates. Specific parameters such as same-day admissions and discharges were noted for accurate length of stay calculations. Ventilation use and mortality were determined using ICD-10 coding and the latter defined as death occurring within 30 days of a COVID-19-related hospitalization.

### Statistical analysis

Our statistical approach involved presenting binary and categorical variables as numbers (percentages), while continuous variables were detailed as medians (with interquartile ranges) and means (with standard deviations). We employed Chi-square tests and ANOVAs using the Tukey method to identify significant differences between the groups. The influence of prediabetes and varying levels of diabetes control on COVID-19-related hospitalizations was explored using multivariable logistic regression. This model incorporated age, gender, and race/ethnicity as independent variables, with hospitalization as the dependent variable. To ensure robustness, we used a bivariate analysis for additional independent variables, with a stepwise selection method for final variable selection. All analyses were conducted using SAS 9.4 software.

## Results

This study reports on 2071 members of KPGA with laboratory-confirmed COVID-19 disease. The socio-demographic characteristics of our population are summarized in **Table 1**. Patients with diabetes and an HbA1c < 7% (53 mmol/mol) and HbA1c ≥ 7% (53 mmol/mol) ≤ 9% (75 mmol/mol) were the oldest (median age 55 years). Normo-glycemic patients without diabetes were the youngest, with a median age of 43 years (**Table 1**). Most of our study population was female (58.5%). Black ethnicity was the most represented race/ethnicity in the study populations (56.7%) and across all groups in the study. The highest percentage of the KPGA members with confirmed COVID-19 resided in the Northeast Metro Atlanta area (36.9%). Patients with diabetes and HbA1c > 9% (75 mmol/mol) resided in areas with the most unfavorable NDI (0.42) compared to the other groups (**Table 1**).

**Table 1:** Socio-demographic characteristics of the study population.

	Glycemic status, no (%)						
Characteristics	All (N = 2071)	Non-DM (n=986)	Prediabetes (n=658)	DM-A1c<7 (n=183)	DM-A1c ≥ 7 ≤ 9 (n= 162)	DM-A1c >9 (n=82)	p-value
Age, median [IQR], years	52 [43-59]	43 [31-52]	52 [43-60]	55 [48-64]	55 [48-61]	53 [43-57]	<0.0001
Gender, No. (%)							<0.0001
Male	859 (41.5)	354 (35.9)	287 (43.6)	88 (48.1)	81 (50)	49 (59.8)	
Female	1212 (58.5)	632 (64.1)	371 (56.4)	95 (51.9)	81 (50)	33 (40.2)	
Race/Ethnicity, No. (%)							0.0006
Black	1175 (56.7)	505 (51.2)	410 (62.3)	102 (55.7)	105 (64.8)	53 (64.6)	
White	469 (22.7)	269 (27.3)	110 (16.7)	44 (24)	34 (21)	12 (14.6)	
Hispanic	162 (7.8)	85 (8.6)	49 (7.5)	14 (7.7)	6 (3.7)	8 (9.6)	
Asian/Pacific Islander	80 (3.9)	36 (3.7)	29 (4.4)	8 (4.4)	4 (2.5)	3 (3.7)	
Other^*^	185 (8.9)	91 (9.2)	60 (9.1)	15 (8.2)	13 (8)	6 (7.3)	
Insurance, No. (%)							
Commercial	1628 (78.6)	810 (82.2)	497 (75.5)	131 (71.6)	124 (76.5)	66 (80.5)	<0.0001
Medicare	225 (10.9)	61 (6.2)	85 (12.9)	44 (24)	28 (17.3)	7 (8.5)	
Self-pay	112 (5.4)	67 (6.8)	29 (4.4)	4 (2.2)	5 (3.1)	7 (8.5)	
Other^†^	106 (5.1)	48 (4.9)	47 (7.1)	4 (2.2)	5 (3.1)	2 (2.4)	
Median Household Income^‡^, No. (%)							0.0001
<50k/year	412 (20.4)	176 (17.8)	136 (20.7)	40 (21.9)	37 (22.8)	23 (28)	
50k-75k/year	1023 (50.6)	460 (46.7)	359 (54.6)	82 (44.8)	82 (50.6)	40 (48.8)	
>75k/year	585 (29)	327 (33.2)	148 (22.5)	54 (29.5)	41 (25.3)	15 (18.3)	
Residential Region^§^, No. (%)							0.0936
Northeast	765(36.9)	376 (38.1)	236 (35.9)	59 (32.2)	62 (38.3)	32 (39)	
Northwest	323 (15.6)	174 (17.6)	92 (14)	24 (13.1)	27 (16.7)	6 (7.3)	
Southeast	426 (20.6)	181 (18.4)	149 (22.6)	45 (24.6)	34 (21)	17 (20.7)	
Southwest	431 (20.8)	199 (20.2)	143 (21.7)	36 (19.7)	29 (17.9)	24 (29.3)	
Neighborhood Deprivation Index^||^	0.15	0.04	0.21	0.22	0.27	0.42	<0.0001
Educational attainment, mean %^¶^							<0.0001
Some High School	6.8	6.6	6.8	6.8	7.1	7.2	
High School Graduate	22.7	22.1	23.2	23.3	23.5	23.5	
Some College	29.4	28.9	29.9	29.6	30.1	29.7	
College Graduate	33.6	34.9	32.6	32.7	31.5	31.7	

^*^ Other race includes Alaska Native/American Indian, unknown and declined to report.

^†^ Other Insurances include military HMO and PPO.

^‡^ ESRI^®^ Business Analyst data is a suite on ArcGIS^®^. ESRI^®^ offers comprehensive demographic and lifestyle segmentation for various geographical levels in the U.S. for identifying underserved communities. The data provided by ESRI^®^ showed median income by zip code, which was then linked to individual patients based on their recorded residence.

^§^ The Atlanta metro area was divided by county, Northwest: Cobb, Cherokee, Paulding, Bartow, Pickens, Polk, Troup, Habersham. Northeast: Dekalb, Gwinnett, Forsyth, Hall, Barrow, Jackson, Butts, Gilmer, Pike, Gordon, Jasper, Monroe. Southwest: Fulton, Douglas, Fayette, Coweta, Carroll, Meriwether, Heard, Dawson, Madison, Lumpkin. Southeast: Clayton, Henry, Rockdale, Walton, Clarke, Spalding, Oconee, Muscogee, Brooks, Town.

^||^ The neighborhood deprivation index is a composite measure of social and economic factors such as income, education, employment and housing quality that reflect neighborhood deprivation and have been linked to adverse health outcomes (Messer et al., 2006). The higher the index, the higher the level of deprivation in the neighborhood.

^¶^ Expressed in mean percentage because it is based on ESRI^®^ Business Analyst data, which provided the count of people’s education attainment and occupation in each zip code by category, and then they provide a denominator by zip code.

### Risk factors and clinical characteristics

Patients with HbA1c > 9% (75 mmol/mol) had the highest proportion of obesity (76.8%), hyperlipidemia (82.9%) and congestive heart failure (17.1%) (**Table 2**). Patients with diabetes and HbA1c ≥ 7% (53 mmol/mol) ≤ 9% (75 mmol/mol) had the highest proportion of hypertension (77.2%), asthma (14.8%), cancer (4.3%), depression (19.8%), more than 2 comorbidities (93.2%) and more than 3 comorbidities (72.2%). They also had the highest mean BMI of 36.4 [8.5] kg/m2. The highest proportions for arrythmia (10.9%) and COPD (6%) were amongst patients with HbA1c < 7% (53 mmol/mol) (**Table 2**).

**Table 2:** Comorbidities in SARS-Cov-2 patients.

	Glycemic status						
Characteristics	All (N = 2071)	Non-DM (n=986)	Prediabetes (n=658)	DM-A1c<7 (n=183)	DM-A1c ≥ 7 ≤ 9 (n= 162)	DM-A1c >9 (n=82)	p-value
Comorbidities/Risk Factors							
Hypertension, n (%)	640 (30.9)	159 (16.1)^a^^,^ ^b^^,^ ^c^^,^ ^d^	169 (25.7)^e^^,^ ^f^^,^ ^g^	126 (68.9)	125 (77.2)	61 (74.4)	<0.0001
Systolic BP, (mmHg), mean [SD]	124.8 [14.5]	121.6 [13.8]^a^^,^ ^b^^,^ ^c^^,^ ^d^	128.2 [14.4]^f^^,^ ^g^	128.8 [16.1]	132.6 [14.1]	132.7 [16.2]	<0.0001
Diastolic BP, (mmHg), mean [SD]	72.3 [11]	71.2 [11.3]^a^^,^ ^d^	73.4 [10.6]	72.4 [11]^i^	73.7 [11.7]	76.8 [10.3]	<0.0001
Obesity (BMI>30), n (%)	1239 (59.8)	470 (47.7)^a^^,^ ^b^^,^ ^c^^,^ ^d^	455 (69.1)	127 (69.4)	124 (76.5)	63 (76.8)	<0.0001
BMI, mean [SD]	33 [7.3]	31 [6.8]^a^^,^ ^b^^,^ ^c^^,^ ^d^	34.5 [7.9]^f^	34.7 [7.3]	36.4 [8.5]	35.4 [7]	<0.0001
Hyperlipidemia, n (%)	776 (37.5)	193 (19.6)^a^^,^ ^b^^,^^d^	259 (39.4)^e^^,^ ^f^^,^ ^g^	130 (71)^i^	126 (77.8)	68 (82.9)	<0.0001
Total Cholesterol, mean [SD]	184.1 [39.1]	183.3 [37]^b^^,^ ^c^	188.2 [38.6]^e^^,^ ^f^	163.9 [38.3]^i^	169.8 [46.6]^j^	194.2 [49.2]	<0.0001
HDL Cholesterol, mean [SD]	53.2 [12.1]	54.9 [14.4]^a^^,^ ^b^^,^ ^c^^,^ ^d^	50.4 [12.5]^f^	46.7 [10.6]	44.5 [11.4]	46.2 [3]	0.0003
LDL Cholesterol [SD]	117.5 [24.4]	113.6 [34.2]^a^^,^ ^d^	123.3 [34.8]^e^	101.4 [41.8]^i^	110.3 [39.2]	128.2 [42]	<0.0001
Triglycerides, mean [SD]	117.6 [12.8]	106.2 [70.3]^c^^,^ ^d^	118.1 [66]^g^	117.6 [55.5]	143.5 [74]	162.4 [98.8]	<0.0001
CAD, n (%)	90 (4.3)	18 (1.8)^b^^,^ ^c^^,^ ^d^	22 (3.3)^e^^,^ ^f^^,^ ^g^	23 (12.6)	19 (11.7)	8 (9.8)	<0.0001
CHF, n (%)	95 (4.6)	18 (1.8)^b^^,^ ^c^^,^ ^d^	19 (2.9)^e^^,^ ^f^^,^ ^g^	22 (12)	22 (13.6)	14 (17.1)	<0.0001
Arrhythmia, n (%)	81 (3.9)	20 (2)^b^^,^ ^c^^,^ ^d^	23 (3.5)^e^^,^ ^f^	20 (10.9)	12 (7.4)	6 (7.3)	<0.0001
Asthma, n (%)	176 (8.5)	77 (7.8)^b^^,^ ^c^	43 (6.5)^e^^,^ ^f^	24 (13.1)	24 (14.8)	8 (9.8)	0.0014
COPD, n (%)	44 (2.1)	9 (0.9)^b^^,^ ^c^^,^ ^d^	13 (2)^e^^,^ ^f^	11 (6)	8 (4.9)	3 (3.7)	<0.0001
HIV, n (%)	18 (0.9)	4 (0.4)^c^^,^ ^d^	6 (0.9)^g^	2 (1.1)	3 (1.9)	3 (3.7)	0.0182
CKD^*^^,^ n (%)	29 (1.4)	6 (0.6)^b^^,^ ^c^	6 (0.9)^e^^,^ ^f^	10 (5.5)	6 (3.7)	1 (1.2)	<0.0001
Depression, n (%)	198 (9.6)	84 (8.5)^a^^,^ ^b^^,^ ^c^^,^ ^d^	35 (5.3)^e^^,^ ^f^^,^ ^g^	34 (18.6)	32 (19.8)	13 (15.9)	0.0013
Cancer, n (%)	45 (2.2)	15 (1.5)^b^^,^ ^c^	16 (2.4)	7 (3.8)	7 (4.3)	0 (0)	0.0412
2+ Comorbidities, n (%)	737 (35.6)	165 (16.7)^a^^,^ ^b^^,^ ^c^^,^ ^d^	190 (28.9)^e^^,^ ^f^^,^ ^g^	158 (86.3)^h^	151 (93.2)	73 (89)	<0.0001
3+ Comorbidities, n (%)	398 (19.2)	48 (4.9)^a^^,^ ^b^^,^ ^c^^,^ ^d^	68 (10.3)^e^^,^ ^f^^,^ ^g^	113 (61.8)^h^	117 (72.2)	52 (63.4)	<0.0001
CCI, mean [SD]^†^	1.9 [1.2]	1.6 [1.1]	1.9 [1.3]	2 [1.2]	2.1 [1.2]	2.1 [1.4]	0.2774
Outpatient Medications, n (%)							
Anti-Hypertensive	535 (25.8)	117 (11.9)^a^^,^ ^b^^,^ ^c^^,^ ^d^	186 (28.3)^e^^,^ ^f^^,^ ^g^	95 (51.9)	93 (57.4)	44 (53.7)	<0.0001
Anti-Asthmatic	393 (19)	178 (18)	125 (19)	40 (21.9)	37 (22.8)	13 (15.9)	0.7183
Anti-Hyperlipidemic	539 (26)	102 (10.3)^a^^,^ ^b^^,^ ^c^^,^ ^d^	160 (24.3)^e^^,^ ^f^^,^ ^g^	112 (61.2)	108 (66.7)	57 (69.5)	<0.0001
Corticosteroids	560 (27)	248 (25.2)^a^^,^ ^b^^,^ ^c^^,^ ^d^	107 (16.3)^e^^,^ ^f^^,^ ^g^	55 (30.1)^i^	42 (25.9)^j^	8 (9.8)	0.0003
Diabetes Medication, n (%)							
Oral	351 (16.9)	9 (0.9)^a^	44 (6.7)^f^^,^ ^g^	108 (59)^h^^,^ ^i^	123 (75.9)	67 (81.7)	0.009
Insulin	142 (6.9)	1 (0.1)^a^^,^ ^c^^,^ ^d^	0 (0)^e^^,^ ^f^^,^ ^g^	22 (12)^h^^,^ ^i^	69 (42.6)^j^	50 (61)	<0.0001
Quality of Care Measures/Lifestyle Behaviors							
BP Control^‡^^,^ n (%)	1773 (85.6)	897 (91.8)^a^^,^ ^c^^,^ ^d^	552 (84)^e^^,^ ^f^^,^ ^g^	143 (79.9)	122 (77.2)	59 (74.7)	<0.0001
HEDIS^§^ nephropathy, n (%)	361 (17.4)	3 (0.3)^b^^,^ ^c^^,^ ^d^	38 (5.8)^f^	131 (71.6)	130 (80.2)	59 (72)	0.009
HEDIS^§^ retinopathy, n (%)	266 (12.8)	3 (0.3)	26 (4)	100 (54.6)	92 (56.8)	45 (54.9)	0.6571
Influenza vaccine, n (%)	953 (46)	403 (40.9)^a^^,^ ^b^^,^ ^c^	319 (48.5)	102 (55.7)	89 (54.9)	40 (48.8)	0.0034
Pneumococcus vaccine, n (%)	125 (6)	35 (3.5)^a^^,^ ^b^^,^ ^d^	41 (6.2)^e^^,^ ^f^^,^ ^g^	23 (12.6)	16 (9.9)	10 (12.2)	<0.0001
Smoking, n (%)	248 (12)	104 (10.5)	87 (13.2)	24 (13.1)	21 (13)	12 (14.6)	0.4221
Average Exercise^||^, (mins), mean [SD]	87.4 [116.1]	98.2 [128.3]^a^^,^ ^c^^,^ ^d^	68.7 [104]	80.7 [135.5]	53.2 [67]	52.5 [84]	<0.0001
EVS: Inactive^¶^, n (%)	760 (36.7)	329 (33.4)^a^	266 (40.4)	70 (38.3)	60 (37)	35 (42.7)	<0.0001

BMI, Body Mass Index; BP, Blood Pressure; CAD, Coronary Artery Disease; CCI, Charlson Comorbidity Index; CHF, Congestive Heart Failure; CKD, Chronic Kidney Disease; COPD, Chronic Obstructive Pulmonary Disease; DM, Diabetes Mellitus; Exercise as a vital sign; HEDIS, Healthcare Effectiveness Data and Information Set.

Significance Levels:

^a^ Non-DM vs Pre-DM.

^b^ Non-DM vs DM-A1c<7.

^c^ Non-DM vs DM-A1c ≥ 7 ≤ 9.

^d^ Non-DM vs DM- DM-A1c>9.

^e^ Pre-DM vs DM-A1c<7.

^f^ Pre-DM vs DM-A1c ≥ 7 ≤ 9.

^g^ Pre-DM vs DM-A1c>9.

^h^ DM-A1c<7 **vs** DM-A1c ≥ 7 ≤ 9.

^i^ DM-A1c<7 **vs** DM-A1c >9.

^j^ DM-A1c ≥ 7 ≤ 9 **vs** DM-A1c>9.

^*^ CKD is classified based on diagnosis reported by the ICD-10 code in the patient’s medical history.

^†^ Charlson Comorbidity Index predicts the 10-year mortality of a patient based on age and comorbidities. Scores are summed to provide a total predictive score. The lowest score of 0 corresponds to a 98% estimated 10-year survival rate. (Charlson et al., 1987).

^‡^ Blood pressure was considered controlled if the most recent reading within the last 12 months had a systolic < 140 mmHg and a diastolic < 90 mmHg amongst patients diagnosed with hypertension.

^§^ HEDIS is a performance improvement tool used by healthcare organizations in the United States. This study used this tool to identify the proportion of COVID-19-confirmed patients with chronic disease successfully controlled specifically for nephropathies and retinopathies.

^||^ Average exercise was collected from self-reported weekly exercise minutes.

^¶^ EVS is based on patient-reported weekly exercise minutes. They are classified as inactive for patients who reported less than 10 mins/week.

Patients with diabetes and HbA1c > 9% (75 mmol/mol) had the lowest proportion of people with adequate blood pressure control (74.7%). They also reported less average exercise, 52.5 (84) mins/week, and they had the highest proportion of patients classified as inactive (42.7%) based on their reported weekly exercise minutes (**Table 2**).

### Clinical outcomes

Of the 2071 patients in this retrospective cohort, 354 were hospitalized (17.1%) and 90 were admitted into the ICU (4.4%) (**Figure 1**). The average hospital and ICU LOS were 8.1 [1.2] days and 11.4 [1.1] days, respectively. Among these, there were 33 patients on mechanical ventilation (1.6%), 26 who were readmitted within 30 days for COVID-related reasons (1.3%) and 27 who died (1.3%) (**Figure 1**). The proportion of hospitalized patients significantly increased as glycemic regulation worsened (p < 0.0001). Normo-glycemic patients without diabetes had significantly lower hospitalization than all other groups (p<0.0001 vs each other group) (**Figure 1**). Patients with prediabetes were hospitalized less frequently than patients with diabetes and HbA1c ≥ 7% (53 mmol/mol) ≤ 9% (75 mmol/mol); p=0.0134 and HbA1c > 9% (75 mmol/mol); p=0.0007. Patients with diabetes and HbA1c < 7% (53 mmol/mol) had lower hospitalization rates than those with HbA1c >9% (75 mmol/mol); p=0.0164. ICU admission followed the same pattern as hospitalization, with normo-glycemic patients without diabetes being the least admitted to the ICU; p=0.0005 vs patients with prediabetes; p=0.0031 vs HbA1c < 7% (53 mmol/mol); p=0.0008 vs HbA1c ≥ 7% (53 mmol/mol) ≤ 9% (75 mmol/mol) vs HbA1c >9% (75 mmol/mol); p<0.0001 (**Figure 1**). Patients with diabetes and HbA1c >9% (75 mmol/mol) had higher ICU admissions than those with HbA1c ≥ 7% (53 mmol/mol) ≤ 9% (75 mmol/mol); p=0.0476, HbA1c < 7% (53 mmol/mol); p=0.0212 and those with prediabetes(p=0.0011). Patients with prediabetes had the highest proportion of mortality in this sample compared to the other groups (p=0.0036) (**Figure 1**). Normo-glycemic patients without diabetes had lower mortality rates than patients with prediabetes (p=0.011) and those with diabetes and HbA1c <7% (53 mmol/mol); p<0.0001. Patients with diabetes and HbA1c < 7% (53 mmol/mol) had a significantly higher proportion of deaths than patients with HbA1c ≥ 7% (53 mmol/mol) ≤ 9% (75 mmol/mol); p=0.0479. For ICU LOS, patients with diabetes and HbA1c < 7% (< 53 mmol/mol) had the lowest mean [SD] days, 6.1 (1); p=0.9572. Normo-glycemic patients without diabetes had the lowest proportion of needing mechanical ventilation (1%), and patients with diabetes and HbA1c > 9% (75 mmol/mol); had the highest (3.7%); p=0.1096 (**Figure 1**).

**Figure 1:**
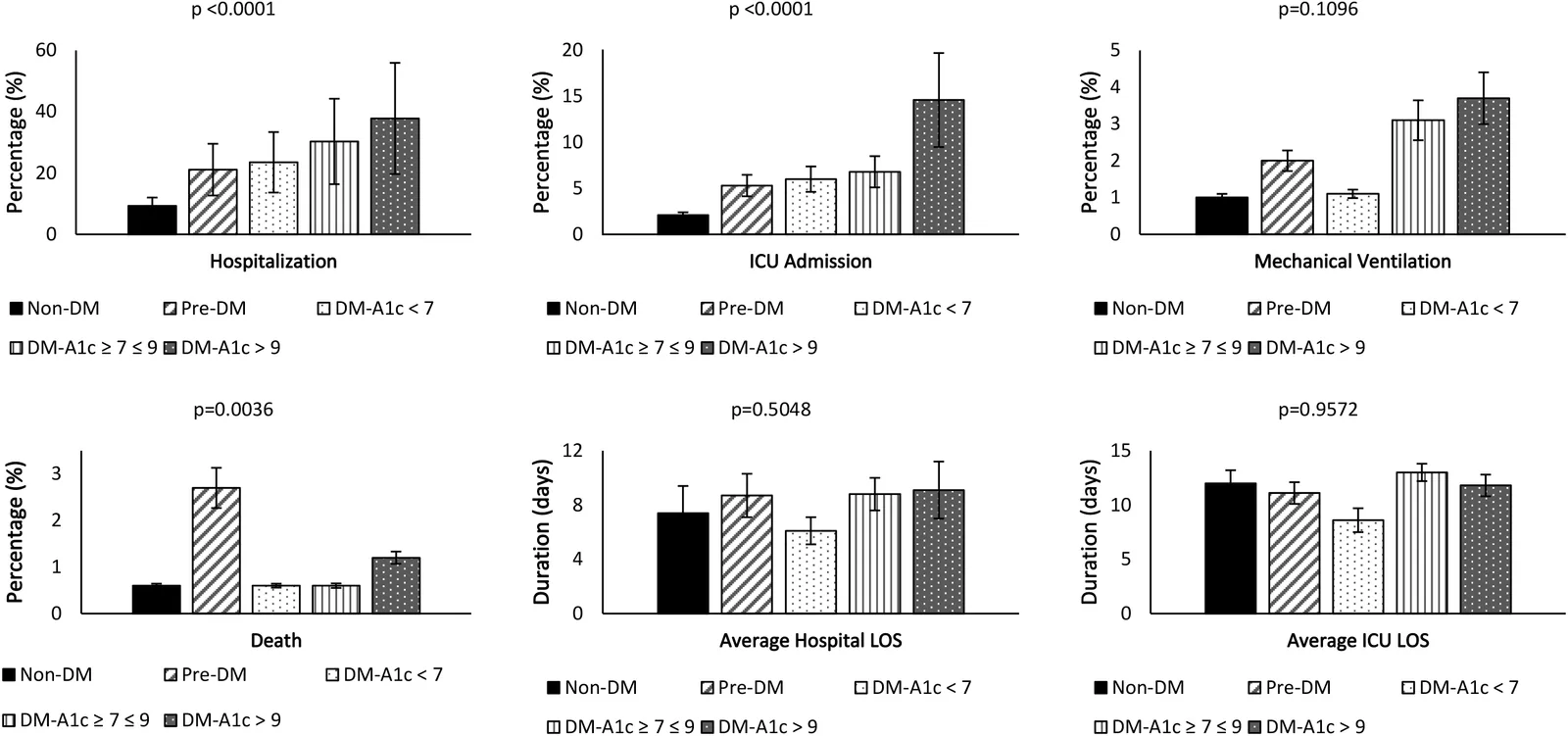
Clinical outcomes amongst COVID-19 patients. Each panel represents an outcome labelled on the x-axis. Hospitalization: Pairwise χ^2^ showed significant differences between normo-glycemic patients without diabetes and all other groups, prediabetes patients and both groups with HbA1c > 7, and patients with HbA1c <7 and those > 9. ICU admission: Pairwise χ^2^ showed significant differences between normo-glycemic patients without diabetes and all other groups, prediabetes patients and both groups with HbA1c > 7, and patients with HbA1c ≥ 7 ≤ 9 and >9. Death: Pairwise χ^2^ showed a significant difference between normo-glycemic patients without diabetes and patients with prediabetes.

### Group odds comparison for hospitalization

When we compared the odds ratios of being hospitalized between glycemic regulation subgroups, we found the following adjusted odds (95% CI): when compared to normo-glycemic patients without diabetes, patients with prediabetes had 1.64 (1.47-1.88) higher odds, patients with diabetes and HbA1c < 7% (53 mmol/mol) had 1.04 (0.59-1.81) higher odds, patients with diabetes and HbA1c ≥ 7% (53 mmol/mol) ≤ 9% (75 mmol/mol) had 2.24 (1.43-3.49) higher odds and patients with diabetes and HbA1c >9% (75 mmol/mol) had 3.3 (1.89-5.78) higher odds of being hospitalized.

Similarly, compared to patients with prediabetes, patients with diabetes and HbA1c < 7% (53 mmol/mol) had an adjusted odds ratio of 0.78 (0.47-1.29), patients with diabetes and HbA1c ≥ 7% (53 mmol/mol) ≤ 9% (75 mmol/mol) had an adjusted odds ratio of 1.35 (0.89 - 2.06), and patients with diabetes and HbA1c > 9% (75 mmol/mol) had an adjusted odds ratio of 2.62 (1.52-4.5) for hospitalization (**Figure 2**). Compared to patients with diabetes and HbA1c < 7% (53 mmol/mol), patients with diabetes and HbA1c ≥ 7% (53 mmol/mol) ≤ 9% (75 mmol/mol) had an odds ratio (95% CI) of 1.3 (0.72-2.33) and patients with diabetes and HbA1c >9% (75 mmol/mol) had an adjusted odds ratio of 1.32 (0.58-3.01) for hospitalization (**Figure 2**). Finally, patients with diabetes and HbA1c > 9% (75 mmol/mol) had an adjusted odds ratio of 1.39 (0.78-2.47) for hospitalization compared to patients with diabetes and HbA1c ≥ 7% (53 mmol/mol) ≤ 9% (75 mmol/mol).

**Figure 2:**
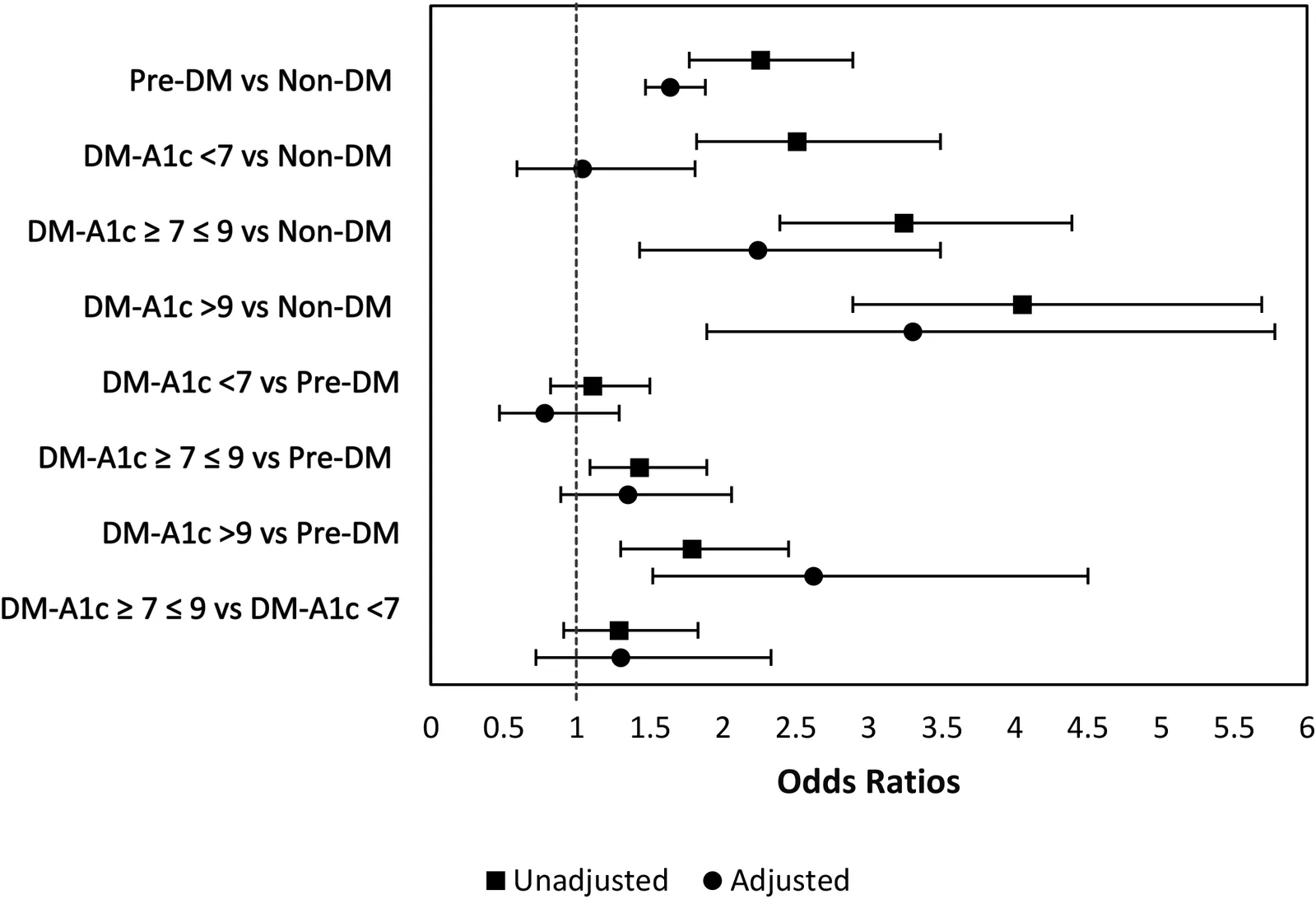
Odds ratio plot showing unadjusted and adjusted odd ratios for hospitalization between study groups. Reference group is listed second in each model after vs. The X-axis shows the odds ratio, and the bars show the 95% CI. The Y-axis shows the two groups being compared.

### Multivariable logistic regression models for hospitalization

In our overall multivariable model, age was associated with greater odds of hospitalization 1.05 (95% CI 1.04-1.06) (**Table 3**). Similarly, smoking 1.72 (95% CI 1.24-2.4), obesity 1.38 (95% CI 1.05-1.81), physical inactivity 1.32 (95% CI 1.03-1.69), prediabetes 1.43 (95% CI 1.09-1.87) and diabetes with HbA1c >9% (75 mmol/mol) 1.84 (95% CI 1.05-3.23) were associated with higher odds of hospitalization (**Table 3**). Residing in the Northeast of Atlanta, and recorded HDL-cholesterol, were associated with lower odds of hospitalization, 0.66 (95% CI 0.5-0.87) and 0.99 (95% CI 0.972-0.999) respectively.

**Table 3:** Multivariable logistic regression model odds ratios for the overall study population and by glycemic status.

	Hospitalization					
Predictors	Overall population (N = 2071)	Glycemic status				
		Non-DM	Pre-DM	DM-A1c < 7	DM-A1c ≥ 7 ≤ 9	DM-A1c >9
Variables OR (95% CI)						
Age	1.05^**^ (1.04-1.06)	1.05^**^ (1.03-1.07)	1.04^**^ (1.02-1.06)			
Female gender			0.64^*^ (0.42-0.97)			
Race Black				3.08^**^ (1.34-7.1)		
Northeast residency^†^	0.66^*^ (0.5-0.87)					
NDI			1.35^*^ (1.03-1.77)			
Commercial Insurance			0.49^*^ (0.3-0.8)			
Median HHI <50k/year					2.66^*^ (1.22-5.8)	
Smoking	1.72^*^ (1.24-2.4)	1.88^*^ (1.05-3.39)				
Systolic blood pressure			1.02^*^ (1.01-1.03)			
Arrhythmia			4.29^*^ (1.49-12.39)			
Coronary artery disease					2.91^*^ (1.05-8.07)	
Congestive heart failure						4.23^*^ (1.22-14.72)
Obesity (BMI>30)	1.38^*^ (1.05-1.81)	2.35^**^ (1.45-3.8)				
Hyperlipidemia				4.04^*^ (1.42-11.51)		
HDL-cholesterol	0.99^*^ (0.972-0.999)					0.95^*^ (0.9-0.99)
Average exercise minutes		0.997^*^ (0.994-0.999)		0.994^*^ (0.989-0.999)		
Physical inactivity^‡^	1.32^*^ (1.03-1.69)					
Insulin use	3.02^**^ (1.95-4.68)			7.4^**^ (2.55-21.45)		
Anti-asthmatic use			2.23^**^ (1.37-3.63)			
Pre-DM	1.43^*^ (1.09-1.87)	n/a^§^	n/a^§^	n/a^§^	n/a^§^	n/a^§^
DM-A1c>9	1.84^*^ (1.05-3.23)	n/a^§^	n/a^§^	n/a^§^	n/a^§^	n/a^§^

HHI, Household Income, OR, Adjusted Odds Ratio, NDI, Neighborhood Deprivation Index.

Significance Levels *p ≤ 0.05; **≤ 0.001.

^†^ NE County Area includes Dekalb, Gwinnett, Forsyth, Hall, Barrow, Jackson, Butts, Gilmer, Pike, Gordon, Jasper, and Monroe counties.

^‡^ Physical inactivity is defined as reported weekly exercise < 10 minutes from the Exercise as a vital sign variable (EVS).

^§^ Pre-DM and DM>9 are not available as independent variables for glycemic status stratified models.

When models were stratified by glycemic control status, age 1.04 (95% CI 1.02-1.06), smoking 1.88 (95% CI 1.05-3.39) and obesity 2.35 (95% CI 1.45-3.8) were associated with greater odds of hospitalization in normo-glycemic patients without diabetes (**Table 3**). Conversely, reported average exercise minutes were associated with lower odds of hospitalization among normo-glycemic patients without diabetes, 0.997 (95% CI 0.994-0.999) (**Table 3**).

Among patients with prediabetes, age 1.04 (1.02-1.06), NDI 1.35 (95% CI 1.03-1.77), systolic blood pressure 1.02 (95% CI 1.01-1.03), arrythmia 4.29 (95% CI 1.49-12.39), and use of anti-asthmatic medication 2.23 (95% CI1.37-3.63) were associated with increased odds of hospitalization whereas, being female 0.64 (95% CI 0.42-0.97) and having commercial insurance 0.49 (95% CI 0.3-0.8) were associated with lower odds of hospitalization (**Table 3**).

Among patients with diabetes and HbA1c < 7% (53 mmol/mol), being Black 3.08 (95% CI 1.34-7.1), having a history of hyperlipidemia 4.04 (95% CI 1.42-11.51) and insulin use 7.4 (95% CI 2.55-21.45) were associated with higher odds of hospitalization while reported average exercise minutes was associated with lower odds of hospitalization (**Table 3**).

Among patients with diabetes and HbA1c ≥ 7% (53 mmol/mol) ≤ 9% (75 mmol/mol), median household income <$50,000 2.66 (95% CI 1.22-5.8) and having history coronary artery disease 2.91 (95% CI 1.05-8.07) were associated with increased odds of hospitalization (**Table 3**).

Among patients with diabetes and HbA1c >9% (75 mmol/mol), having a history of congestive heart failure, 4.23 (95% CI 1.22-14.72) was associated with greater odds of hospitalization, while recorded HDL-cholesterol, 0.95 (95% CI 0.9-0.99) was associated with lower odds of hospitalization (**Table 3**).

## Discussion

This study provides significant insights into understanding the association between varying levels of pre-infection glycemic control and COVID-19 clinical outcomes, including hospitalization, ICU admission, and mortality. Our findings confirm the increased severity of COVID-19 outcomes with increasing HbA1c levels, extending from normo-glycemic patients without diabetes through patients with prediabetes to patients with diabetes and varying degrees of glycemic control. This pattern persisted even after adjusting for various confounders, including socio-economic status, clinical characteristics, diabetes medication, quality of care, and lifestyle behaviors in a diverse population. Age, smoking, obesity, physical inactivity, prediabetes and diabetes with poor glycemic control (HbA1c>9% (75 mmol/mol)) were associated with higher odds of hospitalization. In addition, among patients with diabetes and HbA1c<7% (53 mmol/mol), being Black and insulin use was associated with 3.08 and 7.4 increased odds of hospitalization, respectively. This is particularly important given that approximately 1/3 of U.S. adults have prediabetes (15); these findings have implications for public health strategies, clinical management and targeted interventions for at-risk populations.

### The continuum of glycemic control and COVID-19 outcomes

Our key finding is that worsening glycemic control correlates with increased severity of COVID-19 outcomes, which highlights the need for glucose control in at-risk populations with and without diabetes and priorization for vaccination outreach. Studies show that high blood sugar levels directly increase SARS-CoV-2 replication in human monocytes, and glycolysis allows the continuation of viral replication in the body, which may explain our findings (23). Previous studies have predominantly focused on comparing patients with diabetes versus patients without diabetes or patients with prediabetes versus patients without prediabetes or assessing well-controlled versus poorly controlled diabetes and prediabetes (3, 24, 25). For instance, several studies indicated a higher risk of severe COVID-19 outcomes, such as ICU admissions and mortality in patients with diabetes based on glycemic control (26, 27). Still, they did not differentiate between populations with diabetes and prediabetes.

Individuals with prediabetes, often overlooked in clinical assessments, are known to face a heightened risk of severe COVID-19 outcomes and incident diabetes following infection by SARS-CoV-2 (4). It has also been shown that COVID-19 outcomes worsened in patients with prediabetes based on increasing HbA1c. However, this does not explain the higher percentages of mortality, use of mechanical ventilation and hospital LOS of patients with prediabetes when compared to patients with well-controlled diabetes. This may be because KPGA is an integrated health system; therefore, patients with diabetes may be more closely followed up. In contrast, several patients with prediabetes may be undiagnosed or recently diagnosed based on the national averages (15). Studies suggest that the stress hyperglycemia ratio is a better predictor of mortality and adverse outcomes than abnormal blood glucose in patients with COVID-19, irrespective of their pre-existing chronic glycemic status (28). However, this study highlights that glycemic control before COVID-19 diagnoses may be beneficial, and there is a need for routine clinical management of at-risk groups in the general population to reduce disease severity.

Several studies have not identified pre-infection glycemia as a determinant of COVID-19-related health outcomes. The CORONADO study (29) reported no association between HbA1c and the composite of tracheal intubation or death by day 7 among inpatients with diabetes, whereas BMI and admission plasma glucose were both independently associated with the primary outcome. A comparable finding was obtained in a single-center US cohort of hospitalized patients with diabetes, in which HbA1c quartile was not associated with in-hospital mortality and the authors recommended against the use of HbA1c for risk stratification in that setting (30). Caretto et al. (31) similarly observed no relation between HbA1c and respiratory function among patients hospitalized for COVID-19 pneumonia. These studies differ because patients were enrolled on hospital admission, whereas our study focuses on pre-infection risk factors for outcomes including hospitalization. In addition, enrollment in two of the studies (29, 30) was restricted to patients with diabetes, which excludes the normoglycemic and prediabetes strata across which a substantial proportion of our observed gradient is expressed. A meta-analysis by Prattichizzo et al. (32) examined both exposures within a single evidence base and found that pre-infection HbA1c was associated with COVID-19 mortality, whereas the association was non-significant when the analysis was restricted to studies in which HbA1c was measured at admission. This suggests that pre-infection glycemic status is a risk factor for progression to severe disease requiring hospitalization.

### Role of socioeconomic and demographic factors

As highlighted in our study, the influence of socioeconomic and demographic factors on COVID-19 outcomes echoes the findings of broader social determinants of health research (33, 34). Several studies have shown how factors such as race, income, and access to healthcare significantly influence COVID-19 morbidity and mortality (2, 35, 36). Our study contributes to this literature by linking these socioeconomic and demographic factors to the specific context of glycemic control and COVID-19.

In our regression model, living in Northeast Atlanta, whose counties have consistently higher median income levels, quality housing, green space, better safety and education, was associated with lower odds of hospitalization (37). This corroborates our previous findings, as well as those from other studies, on the role of median income level, housing security, healthcare access, transportation/commuting patterns, and education on COVID-19-related health outcomes (2, 34). This trend is also seen among patients with prediabetes and diabetes (HbA1c ≥ 7% (53 mmol/mol) ≤ 9% (75 mmol/mol), where higher NDI (higher NDI indicates more neighborhood deprivation) and median household income <$50,000 was associated with increased hospitalization, respectively. Conversely, having commercial insurance was associated with reduced odds among patients with prediabetes. This may be due to increased healthcare access due to the insurance type.

Race has been extensively discussed with COVID-19 health outcomes. A recent umbrella review of meta-analyses found that Black and Hispanic individuals had a higher risk of COVID-19 infection and hospitalization compared to White individuals in the pre-hospitalization stage (38). This may be at least partially attributed to higher comorbidity rates in certain ethnic groups (39). Similarly, we found that among patients with well-controlled diabetes, being Black was associated with higher odds of hospitalization compared to their White counterparts.

Concerning SDOHs, our study’s approach aligns with the growing body of literature that advocates for a comprehensive understanding of interacting social determinants of health that extends beyond mere biological factors.

### Comorbidities and COVID-19 severity

Like our findings, numerous studies have reported the exacerbating effect of comorbidities on COVID-19 outcomes. There is a demonstrated higher prevalence and severity of COVID-19 among patients with comorbid conditions, particularly diabetes, hypertension and other cardiovascular diseases, cerebrovascular disease, chronic pulmonary disease, chronic kidney disease and cancer (40). This was supported by a study in the UK that highlighted the increased severity of COVID-19 in patients with comorbidities, even after adjusting for race and ethnicity (41). Our research supports and extends these findings by showing that comorbidities and poor glycemic control (including prediabetes) further increase the risk of severe COVID-19 outcomes, as seen in our regression models.

Integrating comorbidities into our analysis resonates with numerous studies demonstrating the compounded risk multimorbidity poses to COVID-19 patients (42–44). Research by Russell et al. and others has shown that comorbidities and multimorbidity not only exacerbate the severity of COVID-19 but are also associated with the development of post-COVID-19 conditions. Although we observed that patients with diabetes and HbA1c ≥ 7% (53 mmol/mol) a ≤ 9% (75 mmol/mol) had a significantly higher proportion of COVID-19 patients with multimorbidity, there was no association between hospitalization and multimorbidity in our regression model. This may be due to the number of patients with comorbidities across the different study groups. Nonetheless, our study’s focus on the comprehensive profiling of comorbidities allows for a more nuanced understanding of COVID-19 outcomes, particularly in glycemic control and its management.

### Impact of medications on COVID-19 outcomes

The interplay between diabetes medications and COVID-19 outcomes, as found in our study, adds to a growing body of research. Studies have indicated the potential association of at-home insulin use with poor COVID-19 health outcomes (45, 46). Our research contributes to this discourse by examining how various diabetes medications, in the context of different levels of glycemic control, influence COVID-19 severity. The findings from this study corroborate Assaad et al., who found that patients with diabetes on insulin therapy had poorer health outcomes than patients on oral anti-diabetic medication (47). A potential reason for this is insulin, which stimulates the release of cytokines, thereby promoting the cytokine storm observed in severe COVID-19 patients (48). By accounting for the variety and nature of diabetes medications, our study acknowledges their potential impact on glycemic control and the subsequent risk or protection they might offer against severe COVID-19.

Even though asthma does not seem to be an independent risk factor for severe COVID-19 disease, it is important to understand its interaction with anti-asthmatic medications and COVID-19 (49, 50). Severe COVID-19 is associated with significantly elevated cytokines and inhaled corticosteroids (ICS), which are critical for treating asthma and dampening this cytokine storm in the respiratory tract (51, 52). Recent studies exploring the association between asthma medication and COVID-19 outcomes present a nuanced picture (49, 50, 53). While one study on over 7500 COVID-19 patients in South Korea suggests that asthma medication may not independently contribute to adverse COVID-19 outcomes, others highlight the potential influences of ICSs on conferring some degree of protection from SARS-CoV-2 infection and disease severity (49, 50, 53). This underscores the complexity of this relationship, indicating that multiple factors, including chronic and recurrent use of systemic corticosteroids before infection, may increase the risk of poor outcomes related to COVID-19 (50). In our study, the use of asthmatic medication was associated with increased odds of hospitalization in patients with prediabetes. However, we did not distinguish the type of asthmatic medication being used.

### Lifestyle factors

Consistent with our findings, the literature has established a strong link between obesity, physical inactivity, and severe COVID-19 outcomes. Multiple studies have demonstrated that obesity increases the risk of severe COVID-19 independently (54, 55). Our study reinforces this understanding by highlighting obesity’s role in COVID-19 severity in normo-glycemic COVID-19 patients without diabetes, as seen in our regression model. Our focus on obesity as a significant lifestyle factor is supported by extensive research linking obesity to severe COVID-19 outcomes via several mechanisms, including viral entrance, direct viral toxicity, endothelial dysfunction, thromboinflammation, dysregulation of the immune response, and the renin–angiotensin–aldosterone system (56). By incorporating obesity into our analysis, our study acknowledges its dual role as a contributor to poor glycemic control and an independent risk factor for severe COVID-19 outcomes.

Sedentary behaviors and lack of physical activity before COVID-19 diagnosis have been linked to increased risk of severe COVID-19 outcomes (57, 58). Furthermore, it has been associated with post-COVID-19 condition, highlighting the role of physical activity in COVID-19 progression (59). Physical inactivity was an independent risk factor for hospitalization in this study, similar to another study that found that COVID-19 patients who were consistently inactive during the 2 years preceding the pandemic had significantly greater odds for hospitalization, ICU admission and mortality than patients who consistently met physical activity guidelines (57). Subsequent research suggests a dose-dependent association between physical activity and COVID-19 severity, with two key studies in the U.S. (194,00 participants) and South Korea (over 6 million participants) supporting this (58, 60). This association was observed even after adjusting for demographic and clinical characteristics, including race, age, smoking status, BMI, insurance status, and underlying conditions (60). Interestingly, a similar association between moderate to vigorous physical activity and COVID-19 severity is observed even when adjusting for vaccination status (58). A limitation of these studies is the fact that physical activity is self-reported. However, the consistency of these findings shows the need for additional research using more objective measures of physical activity. These findings show the critical role of physical inactivity and sedentary behavior as risk factors not only for chronic diseases but also for severe COVID-19 outcomes.

Smoking was associated with increased odds of hospitalization in both our overall model and in normo-glycemic COVID-19 patients without diabetes. This aligns with the consensus that COVID-19 patients who smoke experience worse disease progression and severity, leading to worse health outcomes (61). Furthermore, a meta-analysis found that former and current smokers had a 30-50% excess risk of COVID-19 progression with patients who had never smoked (62). This underpins the role of public health interventions targeting smoking and other adverse lifestyle factors in preventing severe COVID-19.

### Study strengths and limitations

Our study, while comprehensive, has limitations. Firstly, there is limited power, particularly concerning the number of deaths in our dataset. Secondly, the lack of HbA1c measurements at admission is a significant gap; we relied on the most recent measurements, which could be up to a year old. Consequently, there could have been significant changes in patients’ glycemic control in the interim and thus, potential misclassification of level of glycemic control. Similar data gaps exist for confounders, as most were based on the most recent visit within a year. Furthermore, some of the SDOH data are not at the individual level but rather at the area level, which could limit the precision of our analysis in this aspect. The area level metrics, such as the NDI which uses patients’ reported zip codes, do not reflect individual economic circumstance. Lastly, because this study was done in the context of an integrated health system, these findings may not be as generalizable for populations that have different levels of healthcare access.

However, the strengths of our study are notable. The extent and range of confounders provide a robust analysis of the relationship between glycemic control and COVID-19 outcomes. The continuum of glucose control examined in this study is a unique aspect that adds significant value to the existing literature. Additionally, the diversity of our sample and the inclusion of various SDOHs provide a comprehensive view of the issue. Finally, operating within a closed health system reduces clinical protocol variability, lending credibility to our findings.

In conclusion, our study punctuates the critical role of glycemic control in COVID-19 outcomes and highlights the importance of recognizing prediabetes as a significant risk factor for severe COVID-19. The findings suggest that proactive management and early detection of glucose dysfunction and prediabetes could be crucial in mitigating the risk of severe COVID-19, especially in populations with high rates of obesity and other risk factors. These insights are particularly relevant for healthcare providers and policymakers, who must consider the full spectrum of glycemic control, including blood glucose targets, in their COVID-19 response strategies.

In terms of future research, these results highlight the need for prospective studies with more frequent glycemic monitoring and a detailed analysis of the impact of individual-level SDOHs on COVID-19 outcomes.

## Data Availability

All data relevant to the study are included in the article or uploaded as supplemental information. All data relevant to the study are included in the article. The data analyzed in this study is subject to the following licenses/restrictions: Institutional policy does not permit us to make the datasets publicly available. Requests to access these datasets should be directed to Felipe Lobelo; Felipe.lobelo@kp.org.
